# Magnetic Skyrmions in a Thickness Tunable 2D Ferromagnet from a Defect Driven Dzyaloshinskii–Moriya Interaction

**DOI:** 10.1002/adma.202108637

**Published:** 2022-02-03

**Authors:** Anirban Chakraborty, Abhay K. Srivastava, Ankit K. Sharma, Ajesh K. Gopi, Katayoon Mohseni, Arthur Ernst, Hakan Deniz, Binoy Krishna Hazra, Souvik Das, Paolo Sessi, Ilya Kostanovskiy, Tianping Ma, Holger L. Meyerheim, Stuart S. P. Parkin

**Affiliations:** ^1^ Department for Nano‐Systems from Ions, Spins, and Electrons (NISE) Max Planck Institute of Microstructure Physics Weinberg 2 D‐06120 Halle(Saale) Germany; ^2^ Johannes Kepler University Altenbergerstrβe 69 Linz 4040 Austria

**Keywords:** Fe _3_GeTe _2_, Lorentz transmission electron microscopy, magnetic force microscopy, magnetic skyrmions, non‐centrosymmetric structure, spintronics, van der Waals materials

## Abstract

There is considerable interest in van der Waals (vdW) materials as potential hosts for chiral skyrmionic spin textures. Of particular interest is the ferromagnetic, metallic compound Fe_3_GeTe_2_ (FGT), which has a comparatively high Curie temperature (150–220 K). Several recent studies have reported the observation of chiral Néel skyrmions in this compound, which is inconsistent with its presumed centrosymmetric structure. Here the observation of Néel type skyrmions in single crystals of FGT via Lorentz transmission electron microscopy (LTEM) is reported. It is shown from detailed X‐ray diffraction structure analysis that FGT lacks an inversion symmetry as a result of an asymmetric distribution of Fe vacancies. This vacancy‐induced breaking of the inversion symmetry of this compound is a surprising and novel observation and is a prerequisite for a Dzyaloshinskii–Moriya vector exchange interaction which accounts for the chiral Néel skyrmion phase. This phenomenon is likely to be common to many 2D vdW materials and suggests a path to the preparation of many such acentric compounds. Furthermore, it is found that the skyrmion size in FGT is strongly dependent on its thickness: the skyrmion size increases from ≈100 to ≈750 nm as the thickness of the lamella is increased from ≈90 nm to ≈2 µm. This extreme size tunability is a feature common to many low symmetry ferro‐ and ferri‐magnetic compounds.

## Graphical abstract

The observation of skyrmions in 2D materials has opened a door to novel tunability of their magnetic structure. Here, it is shown that the chiral Néel skyrmions are stabilized in the metallic 2D ferromagnet Fe_3_GeTe_2_, as a result of breaking of the inversion symmetry of its crystal structure that is derived from an asymmetric distribution of Fe atoms in alternate layers in the compound. It is found that the skyrmion size in this compound is strongly influenced by the sample thickness and changes from ≈100 to ≈700 nm as the sample thickness is increased from ≈90 nm to ≈2 µm.

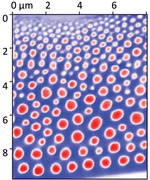

## 1 Introduction

The ever‐growing demand for increased storage density has motivated extensive research in novel spintronic devices.^[^ ^1^ ^]^ Among these the use of magnetic skyrmions, that are nanoscopic spin textures with chiral boundaries, are under consideration as carriers of information.^[^ ^2^ ^]^ Since the first experimental observation of a skyrmion in a single crystal of MnSi in 2009,^[^ ^3^ ^]^ skyrmions have been found in several thin‐film systems^[^ ^4^, ^5^, ^6^, ^7^, ^8^ ^]^ as well as other single crystals.^[^ ^3^, ^9^, ^10^, ^11^, ^12^ ^]^ During this same period, the family of 2 D layered materials has gained significant attention following the successful demonstration of the exfoliation of monolayers of graphene.^[^ ^13^ ^]^ The addition of magnetic van der Waals (vdW) crystals to this family has opened the door to spintronic applications. Bulk crystals of several 2D layered magnetic materials, including Cr_2_Ge_2_Te_6_,^[^ ^14^ ^]^ CrI_3_,^[^ ^15^ ^]^ and Fe_3_GeTe_2_,^[^ ^16^ ^]^ have been shown to display magnetic properties down to thicknesses of just one or a few monolayers. While the first two materials are insulating, Fe_3_GeTe_2_ (FGT) is metallic and, therefore, offers the possibility of the manipulation of spin textures via spin currents. Exhibiting strong perpendicular magnetic anisotropy, together with the possibility of tuning its Curie temperature (*T*_c_) by varying its chemical composition or by ionic gating, FGT is an attractive material for spintronic applications.^[^ ^16^, ^17^, ^18^, ^19^ ^]^

FGT is a metallic layered 2D compound where each layer consists of a Fe_3_Ge slab that is sandwiched between two Te layers. Each of these FGT layers is separated from each other by a vdW gap. FGT is reported to be a ferromagnet with *T*_c_ of about 220 K,^[^ ^20^, ^21^ ^]^ but it has been found that *T*_c_ depends sensitively on small variations in the chemical composition.^[^ ^18^, ^20^ ^]^ Previously, it was reported that this compound has a centrosymmetric structure with the *P*6_3_/*mmc* space group (SGR) (#194). Recently, FGT has also gained attention for hosting magnetic skyrmions, which have a single chirality, and magnetic bubbles, whose chirality is not fixed.^[^ ^22^, ^23^, ^24^, ^25^, ^26^ ^]^ It is claimed that stoichiometric FGT hosts magnetic bubbles rather than skyrmions that require a vector Dzyaloshinskii–Moriya exchange interaction (DMI). Bubbles are consistent with the reported centrosymmetric crystal structure. To account for the observation of skyrmions, it has been argued that the surfaces of the FGT lamellae are either oxidized (e.g., by being exposed to an ambient environment) or must be coupled with a heavy metal, that induces an interfacial DMI which, thereby, stabilizes Néel‐type skyrmions.^[^ ^23^, ^24^, ^25^, ^26^ ^]^ However, the FGT samples that show magnetic bubbles were prepared using either focused ion beam (FIB) or conventional mechanical polishing.^[^ ^22^, ^26^ ^]^ Therefore, the surfaces of these samples would also, very likely, have been oxidized, e.g., during the transfer from the preparation system to the measurements system (in this case, Lorentz transmission electron microscopy (LTEM)). Thus, whether oxidized surfaces accounts for the skyrmions is not clear.

An important assumption that has been made is that the structure of FGT is always centrosymmetric. Using high resolution X‐ray diffraction (XRD) analysis, we have carried out extensive and detailed analysis of the crystal structure of our samples that show Néel‐type skyrmions. We find compelling evidence that the structure of our FGT samples lack inversion symmetry because of i) an asymmetric occupation of lattice sites within alternate Fe_3_Ge layers and ii) due to the statistical occupation of different sites within the vdW's gap by Fe atoms. Density functional theory (DFT) calculations based on the XRD derived structural model provide evidence for the stabilization of a noncollinear magnetic structure via the presence of Fe vacancies and Fe defects in the vdW's gap sites. More details are discussed in the Supporting Information.

## 2 Results and Discussion

The XRD experiments were carried out using a Gallium‐Jet X‐ray source, operated at 70 keV and 200 W, emitting Ga‐Kα radiation (λ = 1.3414 Å) and a six‐circle X‐ray diffractometer. In total 48 reflections were collected on a first single crystal, among them several very weak reflections of type (*H H* 2$\bar{H}$ *L*) with *L* being an odd number which is not compatible with the centrosymmetric SGR *P*6_3_/*mmc* that was previously reported^[^ ^18^, ^20^ ^]^ (for details see the Supporting Information). Quantitative analysis of these data was carried out by a least‐squares refinement of the calculated intensities to those observed based on the SGR #156 (*P*3*m*1). An excellent fit could be achieved which is quantified by the goodness of fit (GOF) parameter of 1.06^[^ ^27^ ^]^ and the unweighted residuum (Ru) of 0.05 where Ru is defined as Ru = Σ||Fobs| – |Fcalc||/Σ|Fobs|, where the sum runs over all reflections. Ru represents the average relative deviation between the calculated (calc) and the experimental (obs) structure factor magnitudes.

**Figure 1:**
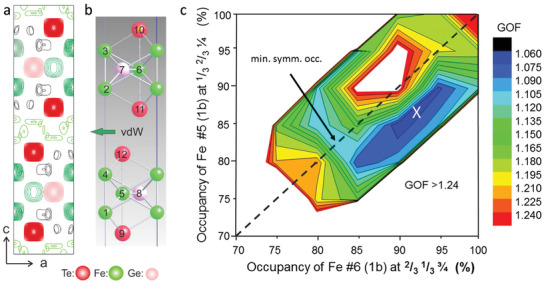
X‐ray diffraction analysis. Charge density ρ(*x*,*y*,*z*) contour plot a) and corresponding structure model b) in projection along the *b*‐axis. Red, green, and pink balls represent Te, Fe, and Ge atoms, respectively. Atoms are labeled in relation to Table S1 in the Supporting Information. Each intense charge density maximum in (a) has a direct counterpart in the refined positions of the atoms. In addition, ρ(*x*,*y*,*z*) also exhibits several faint contours, most prominent within the vdW gap. c): Contour map of GOF versus site occupancies of Fe atoms #5 and #6. The global minimum is indicated by the white cross. The dashed diagonal indicates the condition Θ(5) = Θ(6) in the case of a centrosymmetric space group for which a local minimum at Θ = 0.83 is found.^[^ ^20^ ^]^ The white area corresponds to the condition GOF > 1.24.

**Figure** **1**a,b shows the structural model, together with the calculated charge density ρ(*x*,*y*,*z*) in a projection along the *b*‐axis. Dark red, green, and pink balls correspond to Te, Fe, and Ge atoms, respectively. Atoms are labeled according to Table S1 in the Supporting Information. The most important results are that, apart from the charge density related to the regular sites (#1 to #12), there are a number of faint charge densities within the vdW gap and that the site occupancies (Θ) of Fe atoms #6 and #5 are not identical. For the latter, the precision of the fitting versus the Θ for the Fe sites #5 and #6 is outlined in Figure 1c, which shows a contour plot of GOF versus both site occupancies. The global minimum is indicated by the white cross at ≈ Θ(5) = 0.86 ± 0.03 and Θ(6) = 0.92 ± 0.03. This asymmetry accounts for the reduction in symmetry from *P*6_3_/*mmc* to *P*3*m*1, since in the former case these two sites are equivalent. In Figure 1c, this condition is represented by the dashed line along the diagonal of the plot where a local minimum of GOF is found for Θ(5) = Θ(6) = 0.83. The value is in exact agreement with the refined site occupancy in the previous analysis of an FGT crystal by Deiseroth et al., that was based on the *P*6_3_/*mmc* symmetry.^[^ ^20^ ^]^ In summary, the imbalance of the occupancies of the two under‐occupied Fe sites mostly contributes to the symmetry reduction, i.e., to the loss of the inversion symmetry, while structural relaxations of the atoms along the *z*‐axis play a minor role (<0.05 Å at most). In addition, randomly distributed Fe atoms within the vdW gap (site occupancy ≈5–10% each) also locally destroys the symmetry, albeit on average over the experimental coherence length (≈100 nm) it is restored. More details are given in the Supporting Information. The symmetry reduction to *P*3*m*1 makes the compound a suitable host for Néel skyrmions.^[^ ^28^, ^29^ ^]^ We note that several previous studies have reported the presence of Fe vacancies in the FGT compound,^[^ ^20^, ^30^, ^31^ ^]^ although none of these studies have suggested that these could lead to a non‐centrosymmetric structure, as we find.

To probe the presence of the Fe vacancies indicated by our XRD data, we performed scanning tunneling microscopy (STM) measurements. The FGT crystals are cleaved in situ in the UHV sample preparation chamber of the STM exposing a Te terminated (0001) oriented surface. A typical atomically resolved image acquired using a tungsten tip is shown in Figure S3 in the Supporting Information. Nanoscale fluctuations in the apparent height of the Te atoms is observed, which is consistent with the presence of Fe vacancies under the Te surface, in agreement with our XRD model. Previous STM studies have also proposed Fe vacancies in FGT but, as in our own STM studies, could not reveal the 3D ordering of the Fe vacancies that we find from our XRD data.^[^ ^32^, ^33^, ^34^, ^35^, ^36^ ^]^

**Figure 2:**
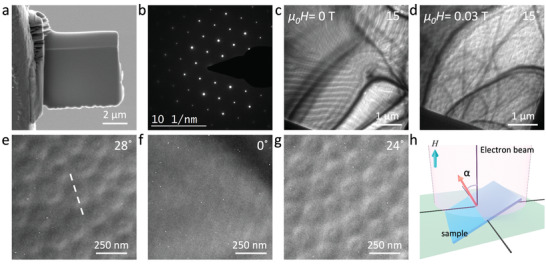
LTEM analysis. a) SEM image of a typical FIBed lamella, b) electron diffraction pattern obtained from the lamella, c) LTEM overview of the lamella recorded at 100 K and zero fields. The sample was tilted 15° away from the [0001] orientation and the image was recorded at a 1.5 mm defocus, d) LTEM image recorded at 100 K after field cooling in a field of 0.032 T, e–g) Néel skyrmions are observed only under tilted conditions. At zero tilt, no magnetic contrast is observed. A defocus value of 1.5 mm is used for the LTEM images. Numbers in the corner of each image gives the tilting angle and the white line in (e) indicates the tilt axis, h) schematic of sample tilting for the LTEM measurements where α is the tilt angle and H is the applied field.

To investigate the magnetic structure of the FGT, LTEM measurements were carried out. The LTEM technique has been widely used for the real‐space observation of magnetic textures such as chiral domain walls, magnetic bubbles, and the ever‐increasing families of skyrmions and anti‐skyrmions.^[^ ^37^, ^38^ ^]^ This technique provides high spatial resolution with the ability to distinguish between different types of magnetic textures.^[^ ^4^, ^9^, ^11^ ^]^ LTEM measurements were performed on different FGT lamellas oriented along the [0001] crystal direction. Lamellae of both uniform thickness and with a variable thickness with a wedge shape were prepared from bulk single crystals of FGT using a FIB milling technique. **Figure** **2**a shows a scanning electron microscope (SEM) image of such a lamella with a uniform thickness that was fabricated from the same crystal used for the XRD data shown in Figure 1. The electron diffraction pattern obtained from the lamella is presented in Figure 2b and confirms that the crystal is [0001] oriented. To observe the magnetic structures, the sample was cooled in the absence of an external magnetic field (zero‐field cooled (ZFC)) from room temperature to 100 K. After the ZFC procedure no contrast could be observed in the LTEM images at a zero tilt angle. However, as the sample was gradually tilted away from the [0001] pole an LTEM contrast, such as is shown in Figure 2c, appeared. A distinct stripe‐like pattern is observed. The appearance of contrast as a result of tilting indicates a Néel‐like chiral spin texture of magnetization. Application of a magnetic field to this zero field state results in the formation of a few skyrmions before they disappear into the field polarized state, as shown in Figure S4 in the Supporting Information. The LTEM images show that only a few well‐separated skyrmions were stabilized using the ZFC procedure. The magnetic contrast in LTEM appears when the image plane is defocused: a defocus value (Δ*f*) of 1.5 mm was found to give the best contrast and was used for all the LTEM images presented here.

It was found that a field cooling (FC) procedure led to an increased number of skyrmions. LTEM studies were carried out over a broad temperature and field window after the sample had been cooled from 300 K (well above *T*_c_) in the presence of a magnetic field of *µ*_0_ *H* = 0.032 T applied along [0001]. A magnetic contrast was observed after tilting the sample away from the [0001] direction. A large number of closely packed skyrmions were observed, as shown in Figure 2d. A magnified image of these skyrmions for different tilt angles is shown in Figure 2e–g. There is no contrast under zero tilt conditions and the contrast reverses when the tilt direction is reversed. The white dashed line in Figure 2e shows the tilt axis. These features provide clear evidence that the observed magnetic structures are Néel type skyrmions.^[^ ^9^ ^]^ A schematic of the sample tilting procedure within the TEM is shown in Figure 2h: the sample tilt angle, α, is defined with respect to the electron beam direction and was varied from zero (plane of sample perpendicular to the beam) to α ≈ ± 27°.

After stabilizing skyrmions using the FC procedure the field was reduced to zero. Skyrmions were found to be stable even after the removal of the field. As the field was then gradually increased to 0.13 T, where the skyrmions disappeared, the diameter of the skyrmions was found to monotonically and substantially decrease. A field polarized state is observed with a further increase in the field. Reducing the field from this state to zero results in a stripe‐like state and no skyrmions are observed at any time during this process. LTEM images recorded during this process are shown in Figure S5 in the Supporting Information. Thus it is clear that the skyrmions stabilized using the FC procedure are metastable skyrmions.

Since the lamella for these measurements were prepared using FIB, it is possible that the surface of the lamella might have been oxidized during the transfer in air to the TEM. Therefore, in order to investigate the effect of oxidation on the magnetic structure, we kept the lamella in an ambient air environment for a period of more than four months. LTEM measurements were performed at different time intervals during this period under the same FC conditions as discussed above. These sequence of LTEM images recorded at different times are shown in Figure S6 in the Supporting Information. These studies showed that the lamella hosts similar Néel type skyrmions and that no apparent change in the skyrmion size or density was observed. This suggests that the change of oxidation state with time (if there is any) does not appear to affect the magnetic textures present in the lamella.

To avoid oxidation of the sample we prepared a encapsulated heterostructure using a different technique, namely, the well‐known dry transfer technique^[^ ^39^ ^]^ with exfoliated flakes from a crystal that came from the same batch as those used in the above studies. In this method, an hBN‐FGT‐hBN sandwich, where hBN is a hexagonal boron nitride flake, was prepared inside a glove box that had an oxygen concentration level lower than 1.5 ppm. This ensures that the FGT surface is protected against oxidation since the FGT surface was not exposed to any solvents during the heterostructure fabrication process. To carry out LTEM measurements, the heterostructure was transferred onto a ≈100 nm thick, electron transparent, silicon nitride membrane. LTEM images recorded at 100 K and for two different tilt angles (α = −27° and +23°) after the same FC procedure is shown in Figure S7 in the Supporting Information. The LTEM contrast is similar to that in Figure 2e,g and reverses upon reversing the tilt direction. These observations show that the magnetic textures observed in the FGT compound are insensitive to oxidation of the lamella surface.

To investigate the effect of the lamella thickness on the skyrmions, measurements were performed on two wedge‐shaped lamellae. The first lamella (L1) with thickness ranging from ≈90 to ≈200 nm was studied with LTEM and the second thicker wedge lamella (L2) with thickness ranging from ≈100 nm to ≈2 µm was studied using magnetic force microscopy (MFM). The same FC and ZFC techniques, as discussed above, were used for the LTEM and MFM studies. Figure S8a in the Supporting Information shows a map of the thickness of the L1 lamella as derived from electron energy loss spectroscopy (EELS). After ZFC, a clear stripe‐like pattern is observed, after tilting the sample away from the [0001], as shown in Figure S8b in the Supporting Information. The contrast again appears only after tilting the sample, thus showing that the stripes have Néel like chirality throughout the entire length of L1. Careful observations reveal that the width of these stripes, i.e., the cycloidal wavelength, increases monotonically with thickness.

**Figure 3:**
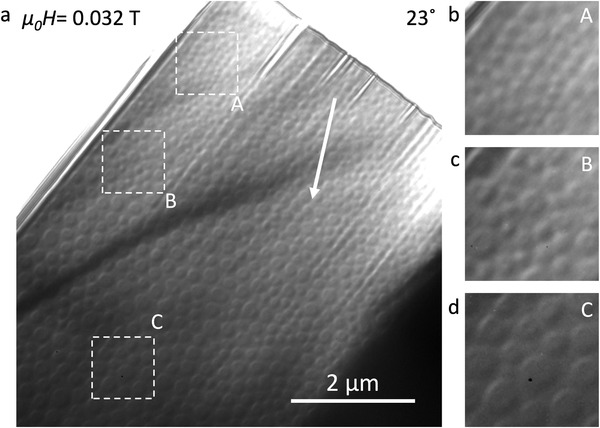
Thickness dependence of skyrmion size in lamella L1. a) LTEM overview of a wedge‐shaped lamella recorded at 100 K after the field cooling procedure described in the main text. Arrow shows the direction of increasing thickness and white squares represent three regions 1 µm × 1 µm in area with different thicknesses labelled as A, B, and C. b–d) magnified images of the three regions shown in (a).

Néel skyrmions were observed in the wedge lamella L1 at 100 K after FC. **Figure** **3**a shows an LTEM overview of the lamella recorded at 100 K with α = 23°. The white arrow shows the direction of increasing thickness. White squares in the image represent three regions of different thicknesses, each with an area of 1 µm × 1 µm. Figure 3b–d shows a magnified view of these three regions. It is evident from these images that the average skyrmion diameter increases with increasing lamella thickness. The skyrmion size was found to decrease with increasing field before finally transforming to the field polarized state just as for the uniform lamellae.

From the LTEM images in Figure 3 one can clearly see that the smaller skyrmions appear as well defined Néel skyrmions, which display a half‐bright and half‐dark contrast silhouette. However, when the skyrmion diameter increases, an additional contrast appears in the center of the silhouette that is consistent rather with a uniformly magnetized central region. Thus the skyrmions look more like chiral Néel bubbles. A simulation of the LTEM contrast for skyrmions with two different diameters is presented in Figure S9 in the Supporting Information. The figure shows how the LTEM contrast changes when the skyrmion core is large enough to be resolved by LTEM.

Although the average skyrmion diameter increases with lamella thickness, a significant variation in the diameter of skyrmions at a given lamella thickness was found. This variation in skyrmion size is likely a result of the FC quenching process itself and the local interactions between skyrmions and their neighbors,^[^ ^6^, ^40^, ^41^ ^]^ rather than local variation in crystalline or magnetic properties of the lamella. No evidence for pinning of the skyrmions to particular sites in the lamella was found.

**Figure 4:**
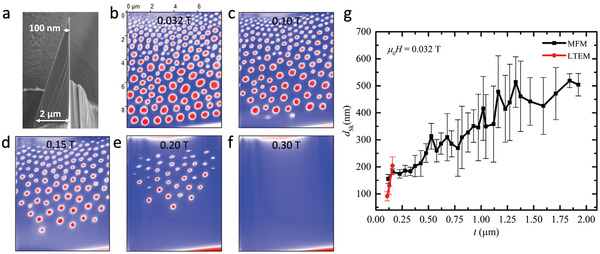
Thickness dependence of skyrmion size in lamella L2 as imaged by MFM. a) SEM image of the wedge‐shaped lamella. The thickness of the lamella varies from ≈100 nm to ≈2 µm. b) MFM image of skyrmions in the lamella at 100 K and 0.032 T. c–f) Evolution of skyrmions as the field is increased from 0.1 to 0.2 T and finally reaches the field polarized state at ≈0.3 T. The blue and red contrast in the MFM images represent up‐ and down‐magnetized domains. All MFM images are at the same scale: a scale bar is shown in (b). g) Skyrmion diameter as a function of lamella thickness including both MFM and LTEM data.

Since LTEM requires transmission of electrons through the sample thickness, we investigated the thicker lamella L2 using only MFM. A side view of this lamella is shown in **Figure** **4**a. We note that the magnetic tips used in the MFM result in imaging only of the out of plane component of the magnetization, in contrast to the LTEM which is sensitive to the in‐plane component of the local magnetization. The ZFC procedure results in stripe‐like patterns or cycloidal states (Figure S10, Supporting Information), whereas the FC results in closely packed circular textures, as shown in Figure 4b. These latter are Néel skyrmions or Néel skyrmion bubbles based on our LTEM studies. One can clearly see that the bubble size, on average, increases with increasing lamella thickness, changing from ≈100 to ≈700 nm, as the thickness is increased from ≈100 to ≈2 µm. The field evolution of the skyrmions is summarized in Figure 4c–f. As the magnetic field is increased from *µ*_0_ *H* = 0.032T the skyrmion size and its stability region gradually shrink, and finally a field polarized state is achieved (Figure 4f). These data are in good agreement with the LTEM observations.

The MFM cannot readily distinguish different types of skyrmions or magnetic bubbles. However, there are close similarities between the Néel type skyrmions observed in LTEM and the magnetic textures observed in MFM, for example, their increased stabilization after FC, a significant size distribution, and a strong thickness dependence of size. Thus, the circular objects observed in MFM are very likely Néel‐like skyrmions that result from the underlying intrinsic DMI of the crystal structure.

In previous reports it has been hypothesized that surface of crystalline flakes or lamellae of FGT were oxidized by exposure to air and that this, thereby, resulted in an interfacial DMI that could account for the observation of chiral Néel skyrmions in an otherwise centrosymmetric compound. Although it is also possible that the surfaces of our FGT lamellae might have been oxidized during their transfer from the FIB to the MFM chamber, it seems unlikely that this could give rise to such a strong interfacial DMI that it could stabilize skyrmions in samples that up to ≈2 µm thick.^[^ ^23^ ^]^ Moreover, in support of our studies that have revealed a bulk DMI arising from a novel mechanism from inhomogeneities in the Fe occupancy that leads to a non‐centrosymmetric structure, skyrmions have been reported in in situ cleaved crystalline FGT samples in an ultrahigh vacuum environment.^[^ ^34^ ^]^ This study and several others have reported that their FGT crystals contained Fe vacancies.^[^ ^32^, ^33^, ^35^, ^36^ ^]^ We would also like to point out that the effects of vacancies and defects have been shown to play a significant role in altering the magnetic properties of various materials.^[^ ^42^, ^43^, ^44^, ^45^ ^]^ Here, our XRD analysis provides, on a quantitative basis, clear‐cut evidence that an imbalance of the concentration of vacancies at Fe lattice sites is primarily responsible for the absence of the inversion symmetry. The observation of skyrmions using LTEM and MFM further supports the non‐centrosymmetric nature of the crystal structure.

## 3 Conclusion

In conclusion, we have unraveled a novel mechanism by which a nominally centrosymmetric 2D ferromagnetic compound can display chiral skyrmions, a property of a non‐centrosymmetric material. The mechanism arises from an imbalance in the occupancy of Fe sites next to the Ge atoms, which we have revealed by X‐ray diffraction studies. Our LTEM studies have clearly shown that these are Néel‐like skyrmions and, moreover, in conjunction with MFM studies, have revealed the very interesting property that the size of these skyrmions in the acentric FGT structure is strongly thickness dependent. This property, that has also been observed in Mn_1.4_PtSn^[^ ^46^ ^]^ and PtMnGa,^[^ ^9^ ^]^ is a consequence of the low symmetry of the DMI vector. Future studies that explore the role of acentricity resulting from excesses or deficiencies of atomic occupancies can lead to novel spin textures.

## 4 Experimental Section

### Lamella Preparation by FIB

Crystals of Fe_3_GeTe_2_ were bought from the company HQ graphene, Inc. and were used for all the measurements presented in this article. For TEM, LTEM, and MFM measurements, several lamellae of different shapes and sizes (uniform thickness and wedge) were prepared using the FIB system TESCAN GAIA 3. The system uses a focused beam of Ga ions for milling and deposition and operates in the range of 0.5 to 30 keV. Lamellae were prepared using the standard lift‐out procedure where Pt was used as a protection layer. After the preparation, the lamellae were polished with a low ion beam energy (3–5 keV) to reduce any damage caused by the ion beam.

To make sure that the observed sample properties are not influenced by Ga ion implantation, two different lamellae were prepared with another FIB system TESCAN FERA 3. This system uses Xe plasma, instead of Ga ions, for FIB procedures. A layer of insulator (SiO*_x_*) was used as a protection for the lamellae prepared in FERA 3. This made sure that the Pt (used in GAIA 3) is also not responsible for any observed properties. A similar polishing procedure with low ion beam energy was performed as the final step.

For MFM measurements, the lamella was transferred on a prepatterned silicon substrate to be easily accessible by MFM tip.

### TEM/LTEM Measurements

TEM/LTEM measurements were performed in FEI‐TITAN 80–300 electron microscope operating at 300 keV. For the LTEM measurements, current in the objective lens of the microscope was modified to provide a perpendicular magnetic field or a field‐free environment to the sample. A double tilt sample holder from GATAN with a liquid nitrogen cooling option was utilized for variable temperature measurements. To measure the thickness profile of the wedge‐shaped lamellae (L1), electron energy loss spectroscopy (EELS) technique was utilized, which uses a log‐ratio method to determine the local thickness (*t*) of the sample using the formula $\frac{t}{\lambda }=\ln\left(\frac{I_{t}}{I_{0}}\right)$, where λ is the inelastic mean free path for the material, *I*_t_ is the total area of spectrum and *I*_0_ is the area under the zero‐loss peak.^[^ ^47^ ^]^

### MFM Measurements

MFM measurements were performed in a variable temperature MFM system (attoLIQUID2000) from Attocube equipped with a vector superconducting magnet that can generate magnetic field both in‐plane and out‐of‐plane to the sample. The measurements were performed in a vacuum. A magnetic tip from Nanosensors (Model: SSS‐MFMR) was used for all measurements. MFM images were recorded using a dual scanning mode. First, the topography of the sample was acquired in tapping mode after correcting the tilt and misalignment of the sample. Then, the MFM tip was lifted by 50 nm from above the sample during the second scan to measure the magnetic signal in non‐contact mode. The frequency shift of the cantilever, caused by the magnetic interactions, was detected using the phase modulation method.

### STM Measurements

STM measurements were performed using electrochemically etched tungsten (W) tips. FGT single crystals were cleaved at room temperature in ultrahigh‐vacuum conditions (*p* = 1e^–10^ mbar) and immediately inserted into the STM head that is operated at liquid nitrogen temperature (*T* = 77 K).

## Conflict of Interest

The authors declare no conflict of interest.

## Supporting information

Supporting Information

## Data Availability

The data that support the findings of this study are available from the corresponding author upon reasonable request.
