# Supplementary material for: Magnetic Skyrmions in a Thickness Tunable 2D Ferromagnet from a Defect Driven Dzyaloshinskii–Moriya Interaction
Source: Adv Mater. 2022 Feb 3;34(11):2108637. doi: 10.1002/adma.202108637 (PMC11475517; doi:10.1002/adma.202108637)
Supplement: Supplementary file 1 — Supporting Information [file ADMA-34-2108637-s001.pdf]

ADVANCED MATERIALS

Supporting Information

for *Adv. Mater.*, DOI: 10.1002/adma.202108637

Magnetic Skyrmions in a Thickness Tunable 2D Ferromagnet from a Defect Driven Dzyaloshinskii–Moriya Interaction

*Anirban Chakraborty, Abhay K. Srivastava, Ankit K. Sharma, Ajesh K. Gopi, Katayoon Mohseni, Arthur Ernst, Hakan Deniz, Binoy Krishna Hazra, Souvik Das, Paolo Sessi, Ilya Kostanovskiy, Tianping Ma, Holger L. Meyerheim, and Stuart S. P. Parkin**

Supporting Information

Magnetic Skyrmions in a thickness tunable 2D Ferromagnet from a defect driven Dzyaloshinskii-Moriya Interaction

Anirban Chakraborty, Abhay K. Srivastava, Ankit K. Sharma, Ajesh K. Gopi, KatayoonMohseni, Arthur Ernst, Hakan Deniz, Binoy Krishna Hazra, Souvik Das, Paolo Sessi, Ilya Kostanovskiy, Tianping Ma, Holger L Meyerheim, Stuart S.P. Parkin[*]

XRD analysis of the bulk crystal

The X-ray diffraction experiments were carried out by using a Gallium-Jet X-ray source operated at 70 keV and 200 W emitting Ga-Kα radiation (λ = 1.3414 Å) monochromatized and focused onto the sample by a Montel optics providing a highly collimated beam of 100 µm in height and 2 mm in width. For data collection, a six-circle x-ray diffractometer was used, where the incident beam was kept constant at an angle of µ = 1 degrees to the surface plane. We used a thin (~100 µm) plate-like shaped Fe_3GeTe_2 (FGT) crystal with lateral dimensions of approximately 2 × 2 mm^2 which at the given X-ray incidence angle was completely illuminated by the X-ray beam.

Integrated reflection intensities were collected by rotating the sample around the surface normal (theta-scan). In total 48 reflections [I(HKL)] were collected reducing to 29 by symmetry equivalence using *P*3*m*1 plane group symmetry. The average agreement between symmetry equivalent reflection intensities is equal to 11%. Figure S1 compares the transverse (theta) scans across the $(01\bar{1}6)$ and the $(11\bar{2}3)$ reflection. While the first is symmetry allowed in SGR $P6_3/mmc$, the latter is forbidden. We have observed other reflections of type (H H $2\bar{H}$ L) as well, e.g. the $(11\bar{2}5)$, $(11\bar{2}9)$, which were also included in the analysis.

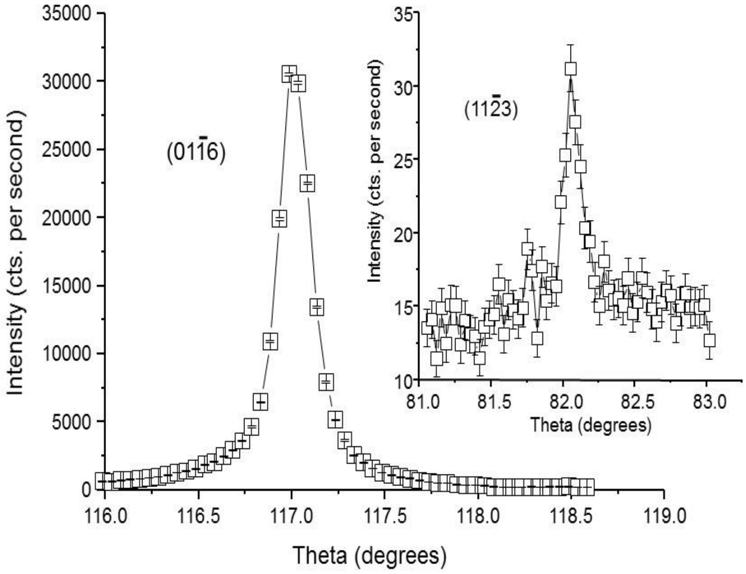

Figure S1. Comparison of theta scans across the (01$\bar{1}$6) and the (11$\bar{2}$3) reflection of single-crystalline Fe_3GeTe_2. Error bars correspond to the 1σ statistical uncertainty. The observation of the (11$\bar{2}$3) reflection is an unambiguous indication that the crystal structure is not completely described by SGR $P6_3/mmc$ (for details see main text).

The observation of these reflections excludes space group $P6_3/mmc$ as well as a number of non-isomorphic subgroups [P3$\bar{1}$c (#163), P6$\bar{2}$c (#190), $P6_322$ (#182), $P6_3mc$ (#186) and $P6_3/m$ (#176)]. For the structure determination, we therefore considered subgroups to $P6_3/mmc$ and among them those for which reflections of type (H H $2\bar{H}$ L) with L odd are allowed for the given Wyckoff sites. Under this condition only space groups $P\bar{3}m1$ (#164), and $P3m1$ (#156) remain as possible choices, the latter one being a subgroup to $P\bar{3}m1$. The distinct difference between these space groups is that SGR $P3m1$ lacks inversion-symmetry.

The best fit was achieved for the structure model based on the $P3m1$ symmetry. We find GOF=1.06 while for a structure model based on $P\bar{3}m1$ GOF=1.43 was obtained, which is

considerably worse. Note, that the GOF also takes into account the number of parameters in relation to the number of reflections.[1] The structure parameters are listed in Table S1

P3m1	x	y	z	U(Å^2)	Occ (%)	GOF
						**1.06**
**(1) Fe 1a**	0.000	0.000	**0.177**	0.025	100	
**(2) Fe 1a**	0.000	0.000	0.674	0.025	100	
**(3) Fe 1a**	0.000	0.000	0.832	0.025	100	
**(4) Fe 1a**	0.000	0.000	**0.330**	0.025	100	
(5) Fe 1b	1/3	2/3	0.250	0.012	86	
(6) Fe 1b	2/3	1/3	0.750	0.012	92	
(7) Ge 1c	1/3	2/3	0.750	0.041	100	
(8) Ge 1c	2/3	1/3	0.250	0.041	100	
**(9) Te 1b**	1/3	2/3	**0.090**	0.027	100	
**(10)Te 1b**	2/3	1/3	**0.910**	0.027	100	
**(11) Te 1c**	2/3	1/3	0.590	0.027	100	
**(12)Te 1c**	1/3	2/3	0.407	0.027	100	
						**0.95**
**(13) Fe 3d**	**0.21**	**0.42**	**0.05**	0.027	7.5	vdW
**(14) Fe 1c**	2/3	1/3	**0.05**	0.027	7.5	vdW

Table S1: Refined coordinates (x,y,z), isotropic atomic displacement parameters (U) and site occupancy factors (*Θ*) in percent of Fe, Te and Ge atoms based on space group *P*3*m*1. Atomic positions are given in relative units with respect to the lattice parameters a = b = 3.94 Å, c = 16.38 Å. Only z-parameters of atoms in bold letters were refined. Atoms in SGR *P*3*m*1 are numbered from (1) to (12) in relation with Figure 1(b) of the main text, where we have discussed that the most prominent result is the asymmetry of the occupancy (*Θ*) of sites (1b)

in SGR $P3m1$. (86 and 92%, respectively). Fe atoms (13) and (14) are located in the vdW gaps by about 7.5% reducing the GOF further from 1.06 to 0.95.

Finally, we comment on the presence of Fe in the vdW gaps which is already evident by the (model-free) faint charge density observable in the contour plot in Figure 1(a) of the main text.

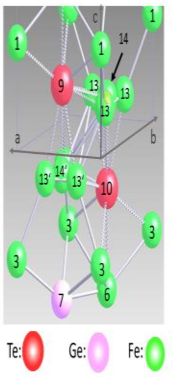

Figure S2: Close-up perspective view of structure model including iron at sites labeled (13) and (14) (see Table S1) Fe atoms labeled by primed numbers (13' and 14') are added to ensure that all vdW gaps contain Fe atoms which would not be the case in SGR $P3m1$.

Different sites were considered according to the charge density contour shown in Figure 2 of the main text and two sites were found to significantly improve the fit quality by up to 10-15% (GOF ~ 0.90). In Figure S2 (a) the structure is shown in a perspective close-up view in the z-range between ±0.25 lattice units. Iron atoms (#13 and #14) occupy vdW sites (see Table1). They are located at Wyckoff sites (3d) (0.42, 0.21, 0.05) for Fe (13) and (1c) (2/3 1/3 0.05) for Fe (14) in SGR $P3m1$. For these atoms, we have added inversion symmetry-related Fe atoms (primed numbers in Figure S2) to ensure the occupancy of all vdW sites by

Fe. Fe atoms #13 and #14 are at a distance of 2.34 and 2.49 Å to the nearest tellurium atoms (uncertainties lie in the 0.1 Å range). The best fit is derived for $\Theta(13) = \Theta(14) \sim 7.5\%$ demonstrating the statistical occupation of different vdW gap sites, which on a length scale smaller than the coherence length of the experiment (~100 nm) completely lifts the crystallographic symmetry.

STM investigations

Figure S3 shows a scanning tunneling microscopy (STM) image of the FGT surface (see Methods for experimental details). As illustrated in Figure 1(b) of the main text, FGT exhibits a crystal structure where the building block consists of a triple-layer Fe_3Ge surrounded on both sides by a layer composed of Teatoms which is nominally perfectly flat. Adjacent Te layers are weakly bound by vdW forces, thus offering a natural cleavage plane that results in a Te-terminated surface. The STM image is taken in a constant current mode so the intensity in the image reflects the variations in the height of the STM tip above the sample surface. The color scale in the image is linear and the dynamic range (low to high) is ±36pm.The STM image is consistent with the expected Te-terminated surface. The Te atoms are clearly seen as the peaks and troughs in the line scans along the dotted white lines in the image: the peak to peak separation of ~0.4 nm is consistent with the Te-Te nearest neighbor separation. Themeasured height corrugation of the Te atoms at the surface, however, varies from place to place. Such variations correspond to fluctuations in the apparent positions of the Te atoms which reflects changes in their electronic structure or slight variations in the positions of the Te atoms away from a nominal flat layer. This can be caused by the presence of defects below the Te surface, in agreement with our XRD model. Such a behavior has been previously reported in FGT, as mentioned in the main text, and in many other layered materials such as magnetic topological insulators, and directly linked to nanoscale fluctuations of the atom positions induced either by dopants or intrinsic defects.[2-3]

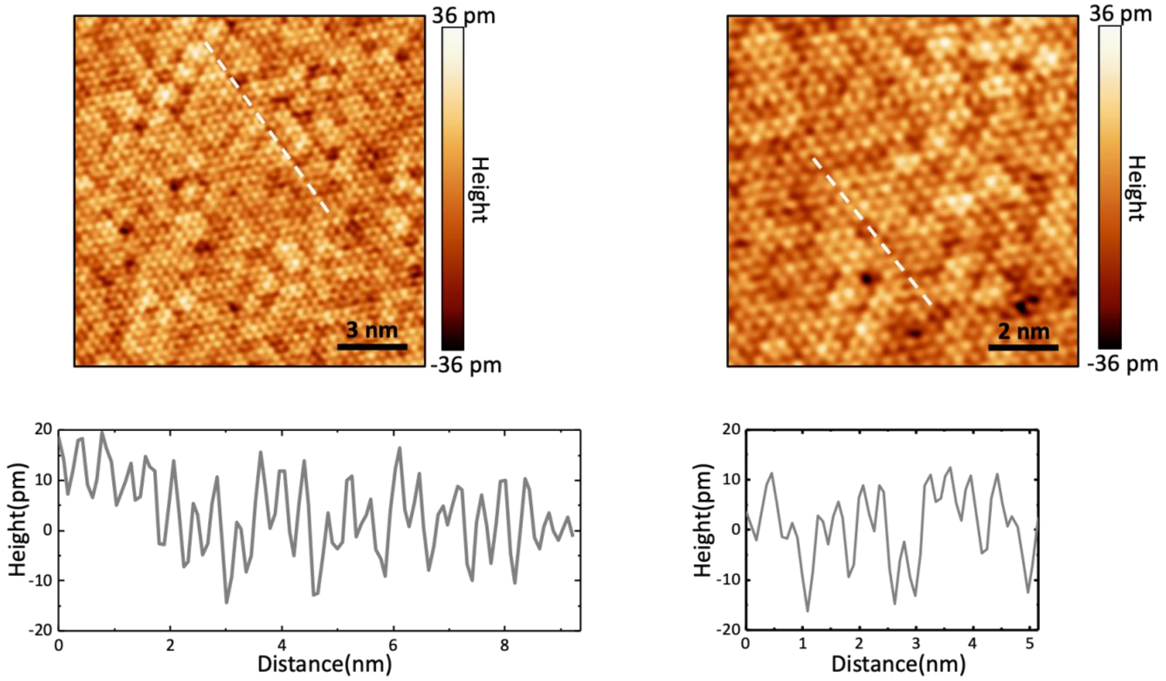

Figure S3: Atomically resolved topographic STM images of FGT recorded at liquid nitrogen temperature after in-situ cleaving of the crystal. The line profiles correspond to the height variations along the white dashed lines in the STM images. The periodic peaks in the line profile correspond to the positions of the Te atoms on the surface.Scan parameters: V= 3 mV, I= 500 pA. The images were analyzed using the WSxM software.[4]

LTEM investigations of FGT

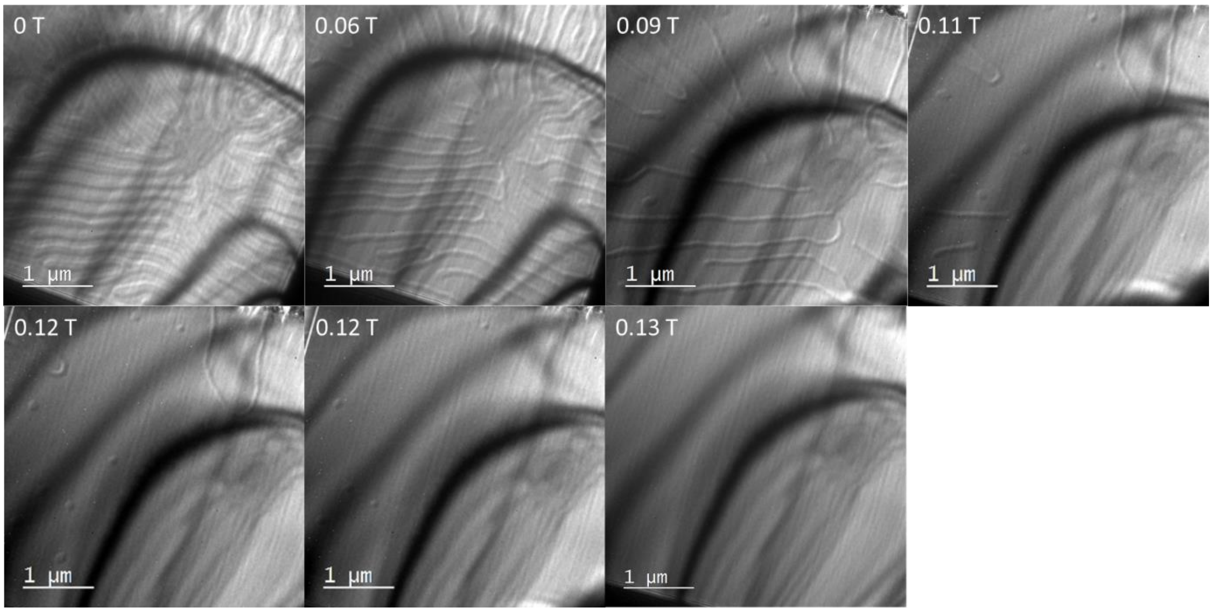

Figure S4: LTEM images are recorded at 100 K under 15° tilted condition and 1.5 mm defocus after the ZFC process.

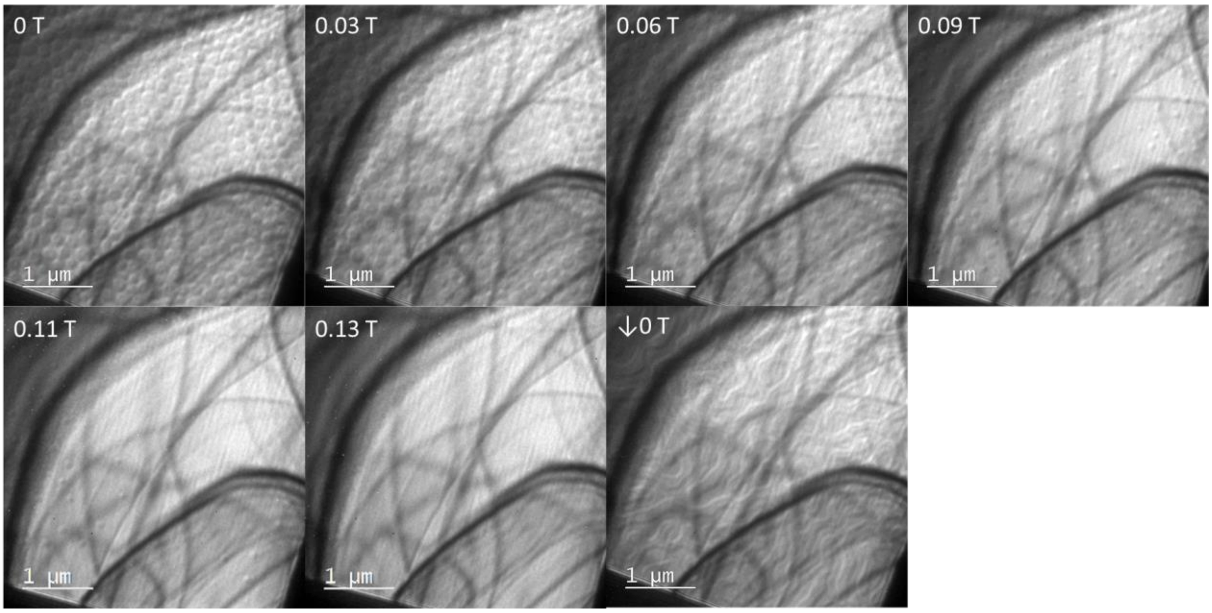

Figure S5: LTEM images are recorded at 100 K under 15° tilted condition and 1.5 mm defocus after the FC process. The downward arrow (↓) in the last image is used to show that after achieving the field polarized state, the field was reduced to zero.

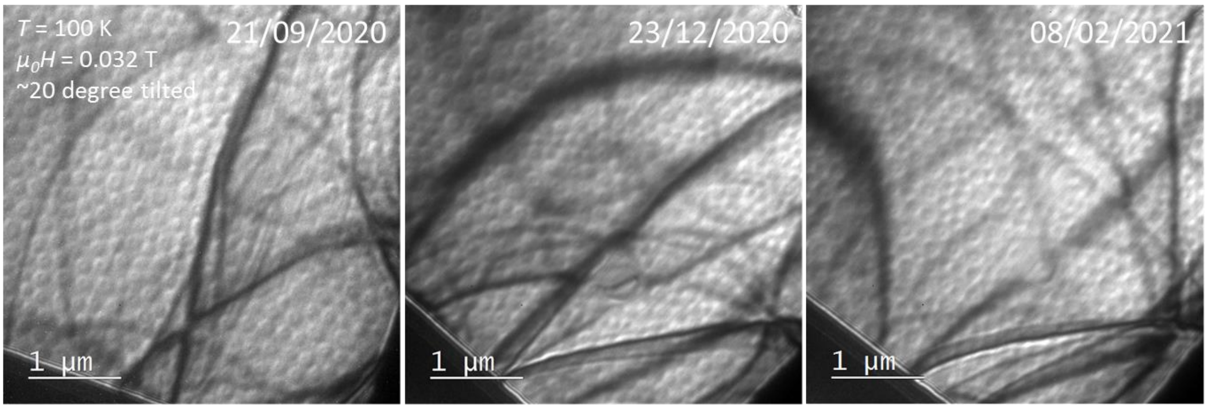

Figure S6: LTEM images recorded on three different dates. The date of recording for each image is shown in the top right corner of the image.

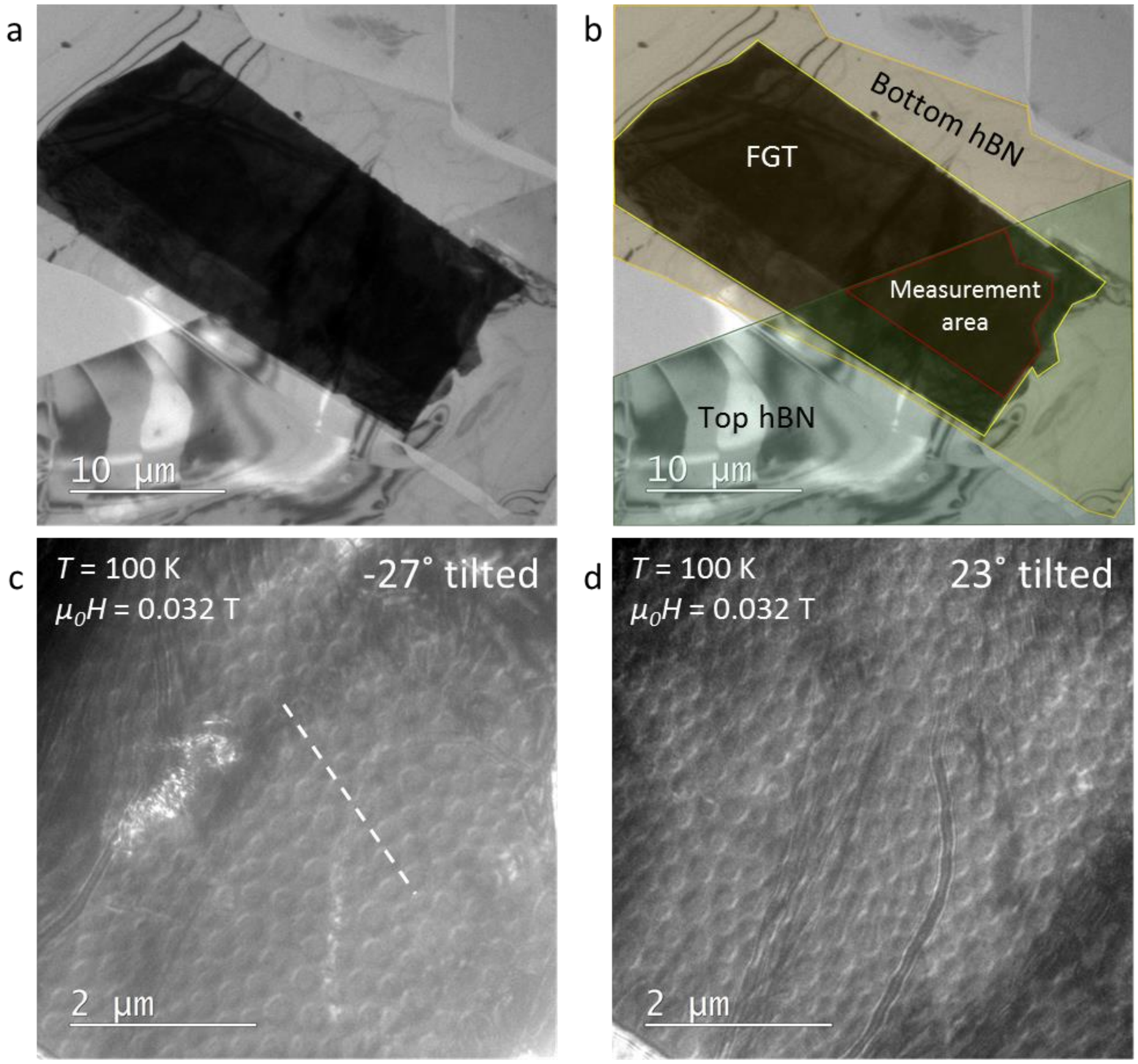

Figure S7:hBN encapsulated FGT flake. (a) Overview of the hBN-FGT-hBN sample, (b) different areas marked by different colors for identification, (c,d) LTEM images under -27° and 23° tilted conditions. The white dashed line in (c) represents the tilting axis.

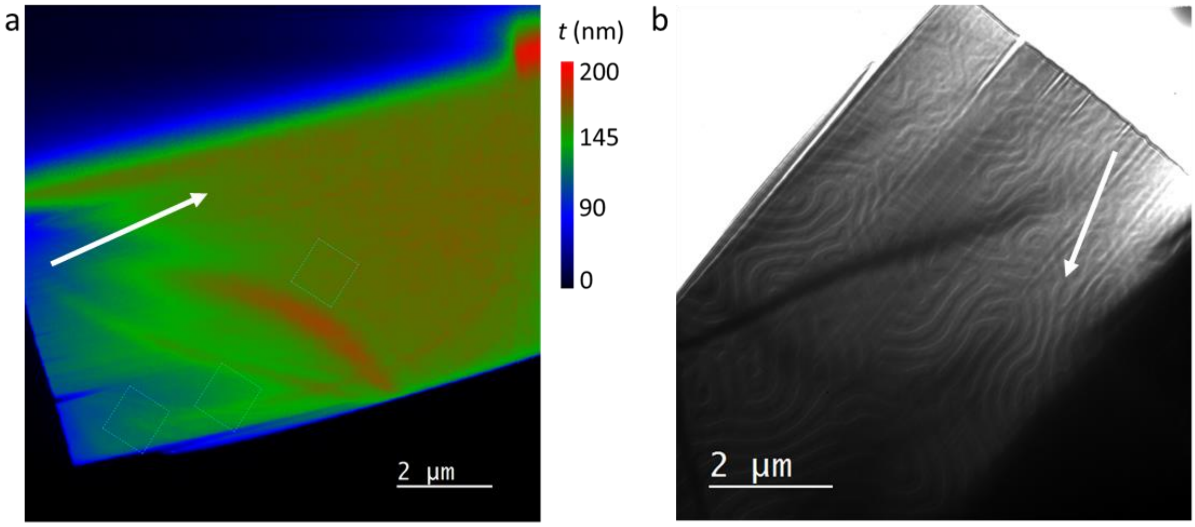

Figure S8: (a) Thickness map of the wedge lamella L1 obtained from EELS measurements where color represents local thickness presented using the color bar. Squares marked in the image are used for measuring skyrmion size at different thicknesses, (b) LTEM overview of the lamella recorded at 100 K and 23° tilt after the ZFC process. The LTEM image is rotated with respect to the EELS image. The white arrows are used to show the direction of increasing thickness.

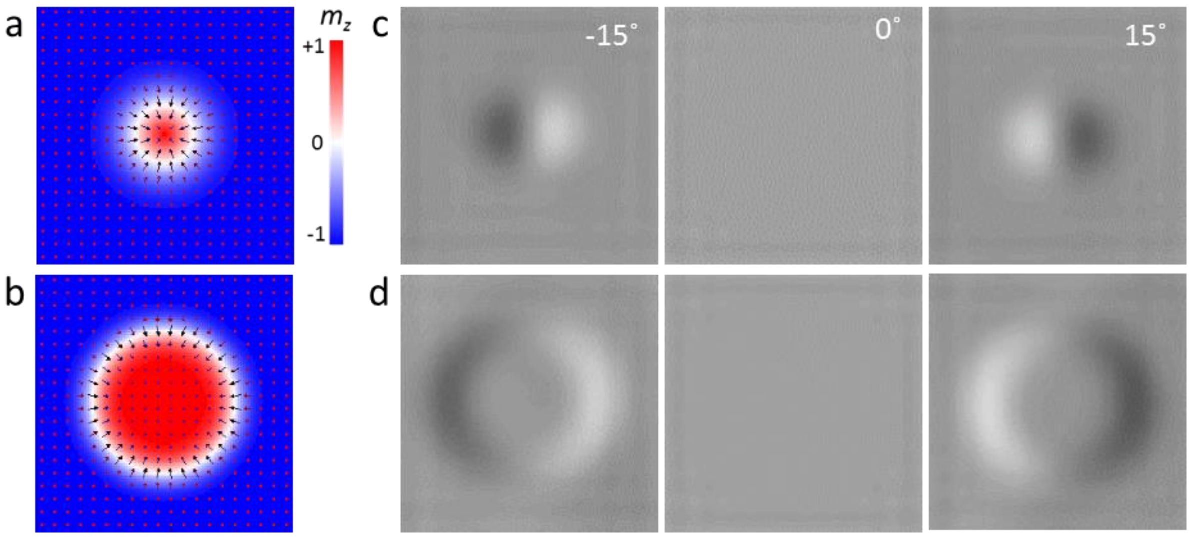

Figure S9: (a,b) Simulated magnetic structure of Néel skyrmions with different diameters and (c,d) corresponding simulated LTEM contrast under -15˚, 0˚ and 15˚ tilted conditions. Micromagnetic simulation software OOMMF[5] with included DMI extension[6] was used to generate magnetization configurations for Néel skyrmion. This configuration was used with MALTS to generate simulated LTEM contrast.[7]

MFM investigations of FGT:

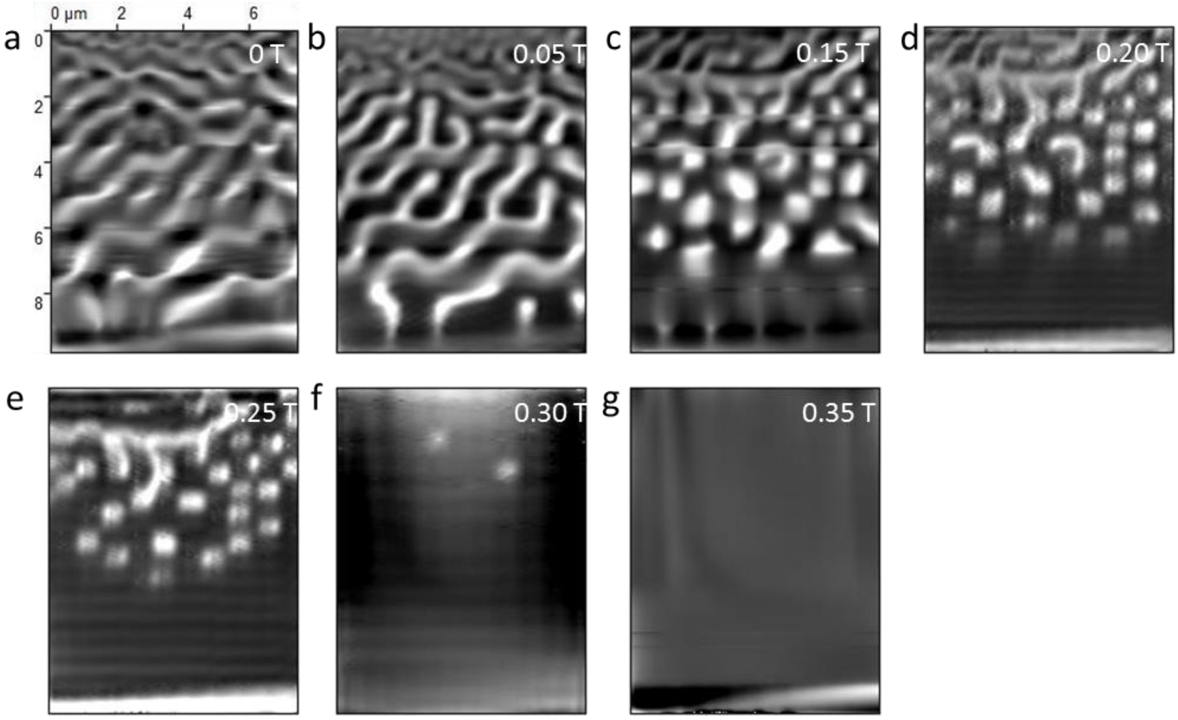

Figure S10: Evolution of domains with magnetic field after ZFC at 100 K. Scale is the same for all MFM images.

Density functional theory (DFT) calculations of the non-collinear magnetic structure in Fe_3GeTe_2

To elucidate further the origin of the skyrmion formation in FGT, we have carried out first-principles calculations of the FGT magnetic structure by comparing the results obtained for the defect-free stoichiometric FGT structure and the structure derived fromour XRD studies

of our FGT crystals. For the calculations we have used a self-consistent Green's function method within the DFT calculations.[8] Disorder effects, Fe vacancies and Fe in the vdW gap, were taken into account within a coherent potential approximation as is implemented within a multiple-scattering theory.[8-9]Localized Fe 3d electron states were treated using a GGA-U approach[10] following a recent theoretical study on FGT.[11]

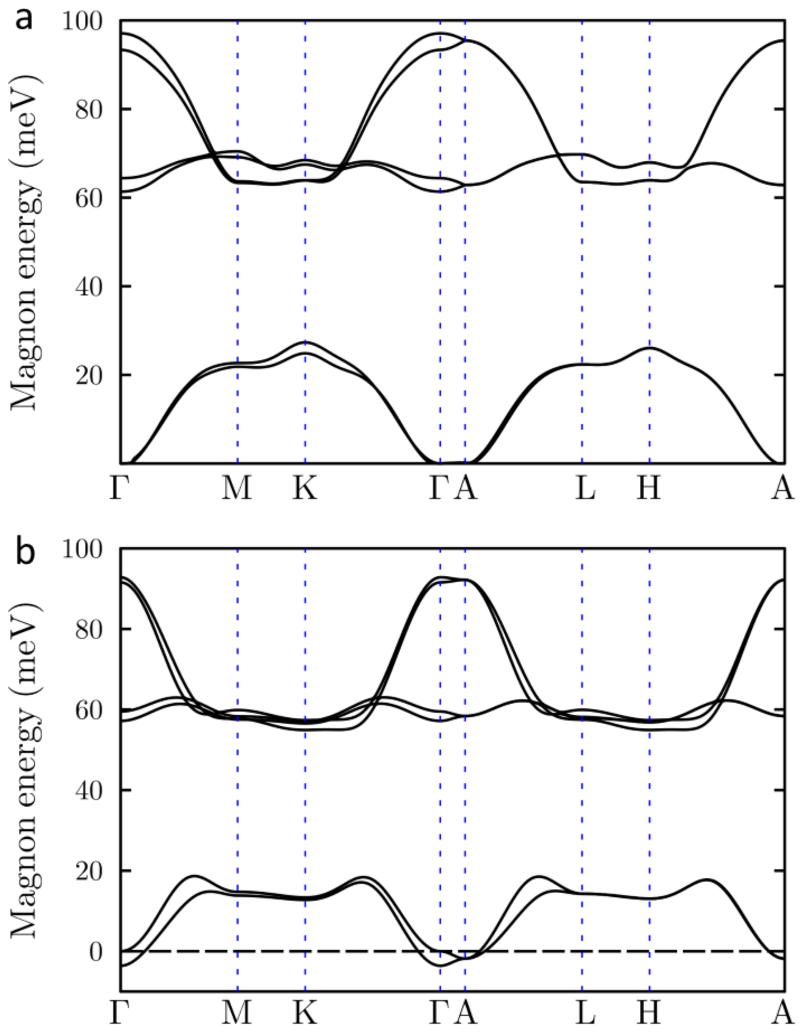

Figure S11: Calculated spin wave spectra for (a) defect-free stoichiometric FGT and (b) FGT with Fe vacancies as in the structure deduced from our XRD measurements.

To describe the magnetic properties of FGT we analyzed spin wave spectra derived by the magnetic force theorem by mapping the DFT electronic structure calculations onto the Heisenberg model.[12]Spin wave (magnon) energies are positive throughout the Brillouin zone

(BZ) in the case of ferromagnetic ordering. If the magnetic order in the system is non-collinear or frustrated, the magnon energies become negative for some q vectors within the BZ. For the defect-free stoichiometric FGT structure the magnon energies are indeed calculated to be positive (see FigureS11 a) confirming the presence of ferromagnetic order. Our calculated magnon spectra are in a good agreement with a recent theoretical prediction.[10]However, the analysis of the magnitude of the exchange coupling parameters in this defect-free FGT structure indicates that it is already close to magnetic instability. The magnetic interaction within the Fe and FeGe planes is dominated by antiferromagnetic coupling, but the overall ferromagnetic order is enforced by the strong ferromagnetic exchange interlayer Fe interaction. Negative magnon energies are found in the vicinity of the Γ- and A- points if: (i) Fe vacancies are introduced in the FeGe planes and (ii) Fe atoms are placed into the vdW's gap in accordance with our structural analysis (see Figure S11 b). This indicates the stabilization of a non-collinear magnetic structure of the FGT in the presence of the aforementioned structural defects, which can be viewed as the origin of the skyrmions in this system.

References:

[1] S. Abrahams, *Acta Crystallogr.***1969**, *A25*, 165.
[2] H. Beidenkopf, P. Roushan, J. Seo, L. Gorman, I. Drozdov, Y. San Hor, R. J. Cava, A. Yazdani, *Nat. Phys.***2011**, *7*, 939.
[3] P. Sessi, R. R. Biswas, T. Bathon, O. Storz, S. Wilfert, A. Barla, K. A. Kokh, O. E. Tereshchenko, K. Fauth, M. Bode, *Nat. Commun.***2016**, *7*, 1.
[4] I. Horcas, R. Fernández, J. Gomez-Rodriguez, J. Colchero, J. Gómez-Herrero, A. Baro, *Rev. Sci. Instrum.***2007**, *78*, 013705.
[5] M. J. Donahue, M. Donahue, *OOMMF user's guide, version 1.0,* US Department of Commerce, National Institute of Standards and Technology, 1999.
[6] S. Rohart, A. Thiaville, *Phys. Rev. B***2013**, *88*, 184422.
[7] S. K. Walton, K. Zeissler, W. R. Branford, S. Felton, *IEEE Trans. Magn.***2013**, *49,* 4795.
[8] M. Hoffmann, A. Ernst, W. Hergert, V. N. Antonov, W. A. Adeagbo, R. M. Geilhufe, H. Ben Hamed, *Phys. Status Solidi B***2020**, *257*, 1900671.
[9] B. Gyorffy, *Phys. Rev. B***1972**, *5*, 2382.
[10] M. Czyżyk, G. Sawatzky, *Phys. Rev. B***1994**, *49*, 14211.
[11] Z.-X. Shen, X. Bo, K. Cao, X. Wan, L. He, *Phys. Rev. B***2021**, *103*, 085102.

[12] A. I. Liechtenstein, M. Katsnelson, V. Antropov, V. Gubanov, *J. Magn. Magn. Mater.***1987**, *67*, 65.
